# Construction of an oxidative stress-related lncRNAs signature to predict prognosis and the immune response in gastric cancer

**DOI:** 10.1038/s41598-023-35167-8

**Published:** 2023-05-31

**Authors:** Hui Zhang, Huawei Feng, Tiansong Yu, Man Zhang, Zhikui Liu, Lidan Ma, Hongsheng Liu

**Affiliations:** 1 School of Life Science, Liaoning University, Shenyang, 110036 China; 2 School of Pharmaceutical Sciences, Liaoning University, Shenyang, 110036 China; 3 Key Laboratory of Computational Simulation and Information Processing of Biomacromolecules of Liaoning Province, Shenyang, 110036 China; 4 Liaoning Provincial Engineering Laboratory of Molecular Modeling and Design for Drug, Shenyang, 110036 China; 5 Key Laboratory for Simulating Computation and Information Processing of Bio-Macromolecules of Shenyang, Shenyang, 110036 China; 6 Liaoning Huikang Testing and Evaluation Technology Co, Shenyang, 110036 China; 7 Dandong Customs Integrated Technical Service Center, Dandong, 118000 China

**Keywords:** Gastrointestinal cancer, Tumour immunology

## Abstract

Oxidative stress, as a characteristic of cellular aerobic metabolism, plays a crucial regulatory role in the development and metastasis of gastric cancer (GC). Long noncoding RNAs (lncRNAs) are important regulators in GC development. However, research on the prognostic patterns of oxidative stress-related lncRNAs (OSRLs) and their functions in the immune microenvironment is currently insufficient. We identified the OSRLs signature (DIP2A-IT1, DUXAP8, TP53TG1, SNHG5, AC091057.1, AL355001.1, ARRDC1-AS1, and COLCA1) from 185 oxidative stress-related genes in The Cancer Genome Atlas (TCGA) cohort via random survival forest and Cox analyses, and the results were subsequently validated in the Gene Expression Omnibus (GEO) dataset. The patients were divided into high- and low-risk groups by the risk score of the OSRLs signature. Longer overall survival was detected in the low-risk group than in the high-risk group in both the TCGA cohort (*P* < 0. 001, HR = 0.43, 95% CI 0.31–0.62) and the GEO cohort (*P* = 0.014, HR = 0.67, 95% CI 0.48–0.93). Next, multivariate Cox analysis identified that the risk model was an independent prognostic characteristic (HR > 1, *P* = 0.005), and time-dependent receiver operating characteristic (ROC) curve analysis and nomogram analysis were utilized to evaluate the predictive ability of the risk model. Next, gene set enrichment analysis revealed that the immune-related pathway, Wnt/$\upbeta$-catenin signature, mammalian target of rapamycin complex 1 signature, and cytokine‒cytokine receptor interaction was enriched. High-risk patients were more responsive to CD200, TNFSF4, TNFSF9, and BTNL2 immune checkpoint blockade. The results of qRT‒PCR further proved the accuracy of our bioinformatic analysis. Overall, our study identified a novel OSRLs signature that can serve as a promising biomarker and prognostic indicator, which provides a personalized predictive approach for patient prognosis evaluation and treatment.

## Introduction

Gastric cancer (GC) is one of the leading causes of cancer-related death worldwide, and these tumors are highly heterogeneous, aggressive, and lethal with nonspecific symptoms, indicating their serious threat to human health^1^. The treatments for GC are multidisciplinary methods based on the patient's clinical and pathological characteristics and usually include surgery, chemotherapy, radiotherapy, and molecularly driven therapy^2^. Although new treatment strategies are continuously proposed for GC, the 5-year survival rate has not yet reached 20–30%. In addition to the abovementioned treatment strategies for GC, immunotherapy has become an emerging treatment method that is only effective for some patients. An increasing number of immune drugs are under clinical research to improve clinical efficiency and decrease the incidence of adverse reactions^3^. Therefore, it is of paramount importance to identify effective diagnostic and therapeutic targets as well as effective biomarkers for evaluating the prognosis of GC patients^4^.

Risk factors associated with GC formation and progression include *Helicobacter pylori* infection, precancerous lesions, and genetic and extrinsic factor^5^. These risk factors regulate the level of reactive oxygen species (ROS) in tumor cells by regulating oxidative stress, which causes the destabilization of cancer-specific genomes^6^. ROS is crucial to regulate cell homeostasis. Moderate ROS levels can promote cell proliferation and regulate cell apoptosis, while high levels of ROS can induce cell apoptosis or necrosis, and cause inflammatory response^7^. Such as ROS can activate FoXO transcription factors, which promotes the expression of certain proteins in cells and promotes the occurrence and development of tumors^8^. Similarly, ROS production is inhibited by cryptotanshinone in cancer cells harboring mutant KRAS, which reduces tumorigenesis^9^. However, oxidative stress has dual effects on tumor immunity, both promoting the initiation of immune responses and possibly inhibiting the persistence of immune responses. For example, an appropriate amount of oxidative stress can induce tumor cell apoptosis, enhance the lethality of immune cells to tumor cells, increase the presentation level of tumor epitopes, etc., thereby promoting the effect of tumor immunotherapy. High levels of oxidative stress are often associated with tumor growth and metastasis, and may affect the efficacy of immune interventions^10^.

Long noncoding RNAs (lncRNAs) are noncoding RNAs more than 200 nucleotides in length that display unique characteristics in regulating cancer proliferation, metastasis, the cell cycle, and programmed death^11^. Previous studies have indicated that lncRNA GABPB1 and its antisense lncRNA GABPB1-AS1 are specifically induced by erastin to regulate oxidative stress in liver cancer cell ferroptosis^12^. Similarly, lncRNA LAMTOR5-AS1 regulates the level of intracellular oxidative stress by controlling the interaction between NRF2 and KEAP1^13^. LncRNA CASC11 inhibits cellular apoptosis and accelerated the cell cycle by sponging miR-340-5p to upregulate CDK1 expression in GC^14^. The lncRNA PINK1-AS promotes Gαil-driven GC progression by sponging miR-200a^15^. Moreover, metabolic plasticity mediated by MACC1-AS1 in the AMPK/Lin28 pathway is a determinant of GC cell growth and metastasis^16^. These studies imply that CASC11 and PINK1-AS may become novel tumor biomarkers. However, the potential of oxidative stress-related lncRNAs (OSRLs) as therapeutic targets for GC has not been fully researched. Therefore, further study of OSRLs is needed to provide new ideas and perspectives for GC research and immunotherapy.

In this study, an OSRLs prediction signature was constructed by a new model combination algorithm, and its performance in predicting prognosis, chemotherapy sensitivity, and cell immune infiltration level was evaluated in GC patients. Overall, our study provides a novel perspective for the treatment of GC and could help clinicians make management decisions.

## Materials and methods

### Patients and datasets

**Figure 1:**
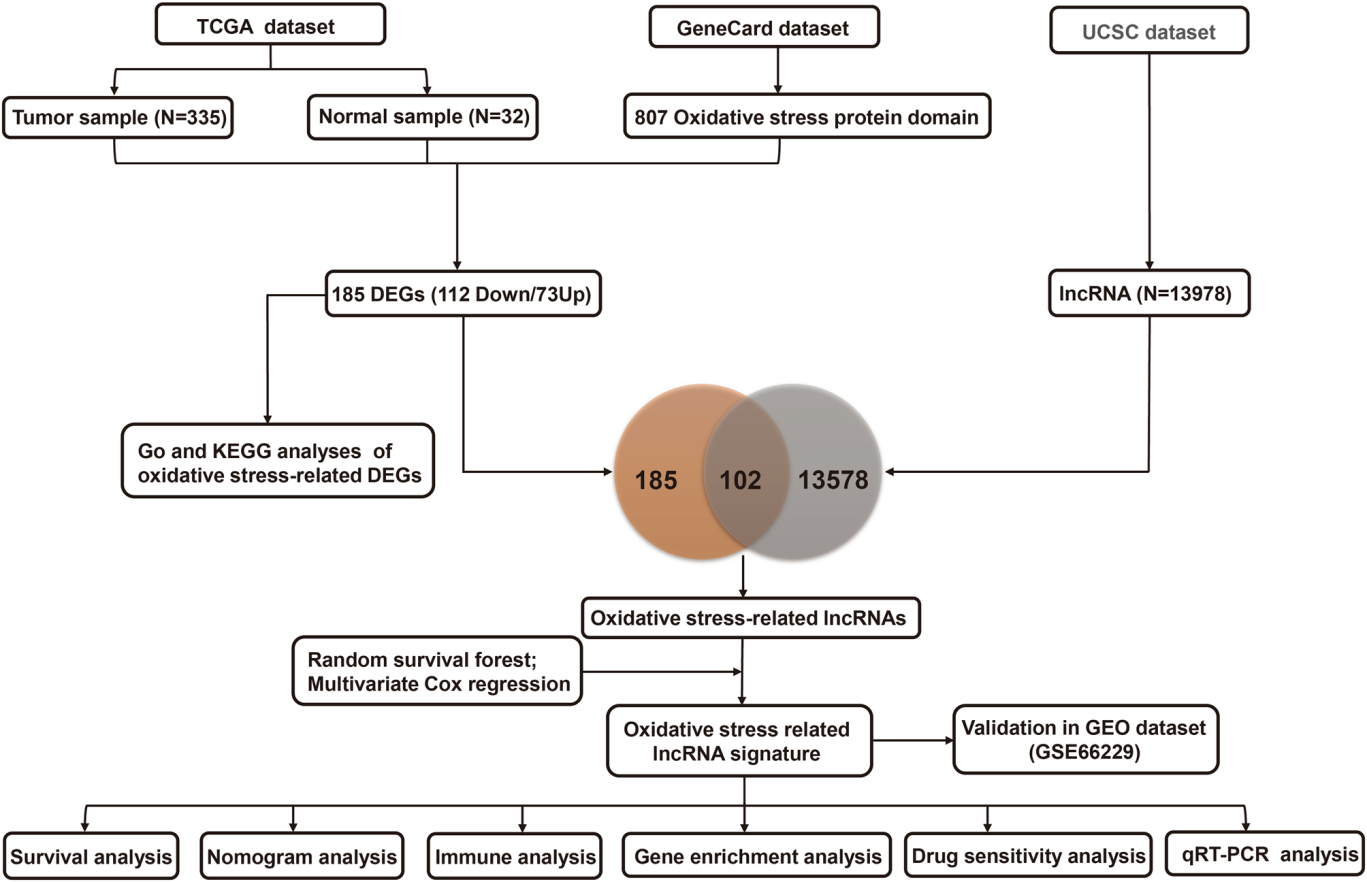
The flowchart of this study. *TCGA* The Cancer Genome Atlas, *UCSC* University of California Santa Cruz, *GEO* gene expression omnibus, *DEGs* differentially expressed genes, *lncRNA* long noncoding RNA, *GO* gene ontology, *KEGG* kyoto encyclopedia of genes and genomes, *qRT‒PCR* quantitative real-time polymerase chain reaction.

The data were assembled from The Cancer Genome Atlas stomach adenocarcinoma (TCGA-STAD), Gene Expression Omnibus (GEO) (GSE66229), and GeneCards datasets. The training set was collected from the TCGA dataset that included RNA-seq data and corresponding clinicopathological characteristics from 375 tumor tissues and 32 normal gastric tissues. Among STAD patients, twenty patients were excluded whose survival time was less than one month and had missing data to ensure the accuracy of prediction. GSE66229 (n = 400), in which the follow-up for patients described exceeded 5 years, was used as an independent validation set. The oxidative stress protein domains (n = 807) with relevance scores higher than 7.0 were obtained from the GeneCards database (Supplementary Table 1), and symbols of human lncRNAs were acquired from the University of California Santa Cruz (UCSC) dataset. Finally, RNA-seq and clinical data were obtained from 367 patients in our OSRLs signature study with the workflow in Fig. 1.

### Oxidative stress-related genes and functional enrichment

The R “limma” package was utilized to confirm 185 oxidative stress-related differentially expressed genes (DEGs) between the GeneCards dataset and the TCGA-STAD dataset according to the thresholds of false discovery rate < 0.05 and |FC|> 2. Gene Ontology (GO) and Kyoto Encyclopedia of Genes and Genomes (KEGG) enrichment analysis were performed with the “clusterProfiler” package.

### Construction and validation of the OSRLs predictive signature

The interaction among oxidative stress-related DEGs and lncRNAs was extracted by the “limma” package. The screening criteria were correlation coefficient |*R*^*2*^|> 0.2 and *P* < 0.001. Afterward, a random survival forest (RSF) algorithm was applied via the “random Forest SRC” package to acquire the OSRLs in GC patients. Next, multivariate Cox regression was exploited to construct an OSRLs predictive signature via the “survival” package. The prognosis-related risk score of each GC patient was calculated as follows:

$$risk \,Score=\sum_{i=1}^{n}({Coef}_{i}\times {x}_{i})$$

${Coef}_{i}$ and ${x}_{i}$ represent the corresponding coefficients and the expression levels of lncRNAs, respectively. For survival analysis, the median value of the risk score was used as the optimal cutoff point to classify GC patients into a high-risk group (HRiG) and a low-risk group (LRiG). The KM method was applied to assess the survival rate of the HRiG and LRiG of GC patients through the R “survival” and “survminer” packages. Moreover, the GSE66229 dataset was estimated with the same risk formula and median risk scores as the training set to assess the predictive ability of the OSRLs signature. Independent predictive characteristics among the OSRLs signature and clinicopathological variables (age, sex, clinical stage, tumor-node-metastasis (TNM) stage, and risk score) were identified by Cox regression analysis in the "survival" package. The R “rms” package was exploited to construct a prognostic nomogram to evaluate the survival rate of GC patients. Subsequently, the calibration curve was used to predict performance in the prognostic nomogram.

### Enrichment analysis and tumor immune microenvironment analysis of the OSRLs prediction signature

To determine the underlying molecular mechanisms and functional pathway of the OSRLs signature, the “GSEA” package was utilized to analyze the degree of enrichment in different pathways between the HRiG and LRiG, and the “CBNplot” package was used for visualization^17^. The “ssGSEA” package used a single sample gene set to evaluate 16 immune infiltration cell scores and 13 immune function scores. The half-maximal inhibitory concentration (IC_50_) was calculated to reflect the sensitivity of cells to conventional chemotherapeutic drugs through the Wilcoxon signed-rank test in GC treatment.

### Cell culture and qRT‑PCR

A human gastric epithelial cell line (GES-1) was purchased from EK-Bioscience (Shanghai, China), and STAD cells (AGS, HGC-27) were purchased from Bio-FIREFLYGLO (Beijing, China). The quality of the three cell lines was certified by short tandem repeat (STR). GES-1, AGS, and HGC-27 cells were cultured in DMEM (L110KJ, Shanghai, China), DMEM/f12 (MA0214, Dalian, China), and RPMI 1640 (MA0215, Dalian, China) complete medium containing 10% fetal bovine serum and 1% double antibody under a humidified atmosphere of 37 ℃ and 5% CO_2_. The UNIQ-10 Column TRIzol Total RNA Isolation Kit (Sangon Biotech, China) was utilized to extract the total RNA from cell samples. Maxima Reverse Transcriptase (Thermo Scientific, China) was used to reverse transcribe RNA to cDNA. Primers for AC091057.1, AL355001.1, ARRDC1-AS1, COLCA1, DIP2A-IT1, DUXAP8, TP53TG1, and SNHG5 (Supplementary Table 2) for qRT‒PCR were synthesized by Sangon Biotech (Sangon Biotech, China). lncRNA expression was performed with 2 × SG Fast qPCR Master Mix (High Rox) using SYBR Green I (BBI, China), and each lncRNA expression level was collected and quantified by the 2^(-∆∆Ct)^ method. All experiments were repeated three times independently.

### Statistical analysis

Statistical analyses were performed by utilizing R 4.2.0. The Wilcoxon test was used to analyze the expression differences between the LRiG and HRiG. The KM method and log-rank test were employed to compare overall survival between subgroups. Cox analysis was utilized to evaluate the prognostic value of variables in GC patients. The differences in the infiltration levels of different immune cell subgroups were assessed via the Mann‒Whitney test. All statistical analyses were two-sided, and the statistical significance threshold was *P* < 0.05.

## Results

### Enrichment analysis of oxidative stress-related genes

**Figure 2:**
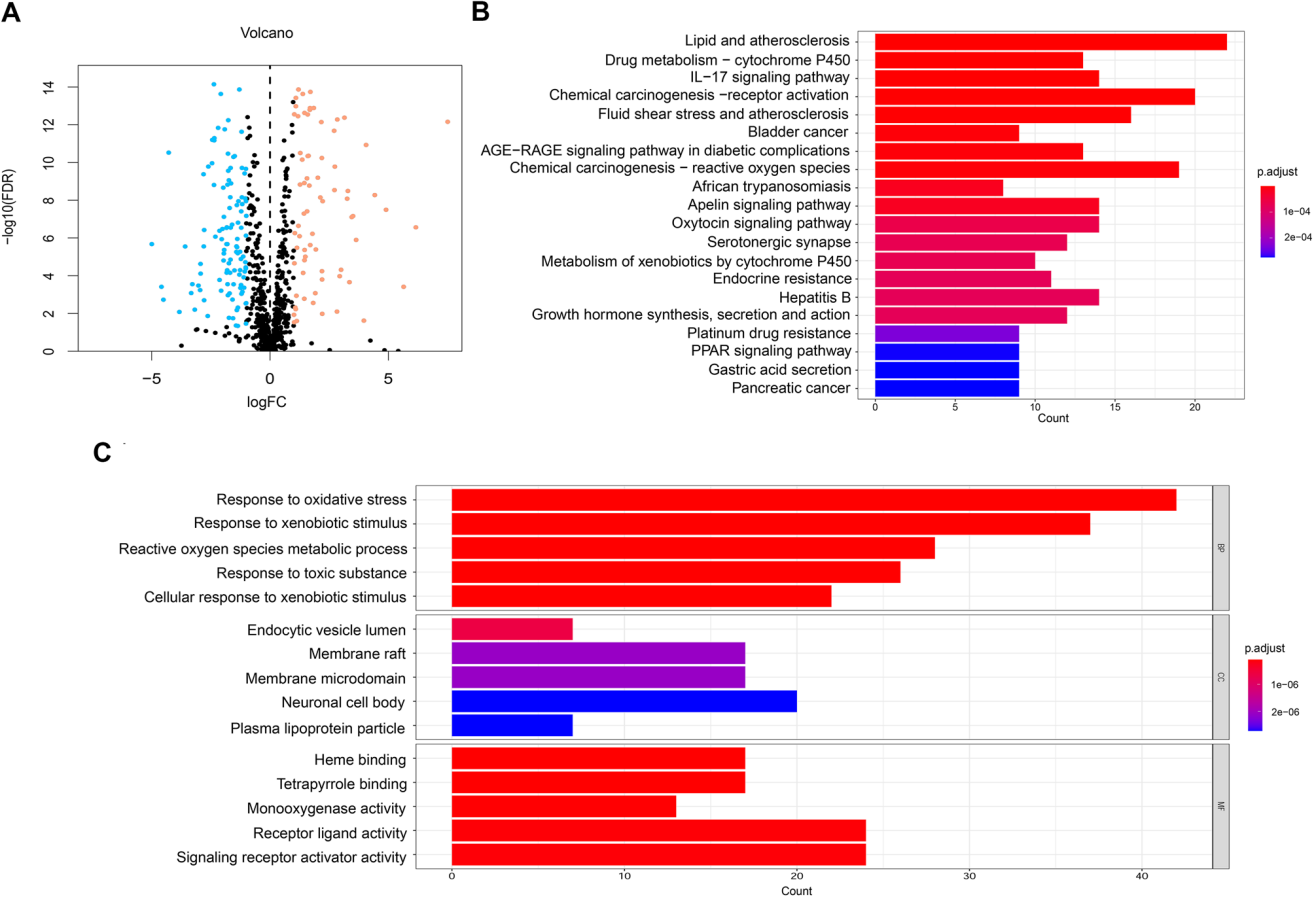
Identification differentially expressed genes of oxidative stress-related genes and functional enrichment analysis. (**A**) Volcano plot of 185 oxidative stress-related DEGs between the normal and GC groups. Blue dots are down-regulated genes, and orange dots are up-regulated genes. (**B,C**) Kyoto Encyclopedia of Genes and Genomes analysis and Gene Ontology analysis of oxidative stress-related differentially expressed genes. The significance of the gene gradually increases from blue to red.

A total of 185 oxidative stress-related DEGs (73 upregulated genes and 112 downregulated genes) were identified in the TCGA-STAD dataset (Fig. 2A and Supplementary Table S3). We then performed KEGG analysis (Fig. 2B and Supplementary Table S4) and GO analysis (Fig. 2C and Supplementary Table S5) to identify the oxidative stress-related DEGs. The top 5 pathways of KEGG analysis were lipid and atherosclerosis, drug metabolism-cytochrome P450, IL-17 signaling pathway, chemical carcinogenesis-receptor activation, fluid shear stress, and atherosclerosis. The GO analysis included biological process (BP), cellular component (CC), and molecular function (MF) categories. In the BP category, DEGs were enriched in the response to oxidative stress, xenobiotic stimulus, and ROS metabolic process. In the CC category, DEGs were associated with endocytic vesicle lumen, membrane raft, membrane microdomain, etc. In the MF category, DEGs were associated with receptor ligand activity, signaling receptor activator activity, etc.

### Identification and validation of the OSRLs prognostic signature

**Figure 3:**
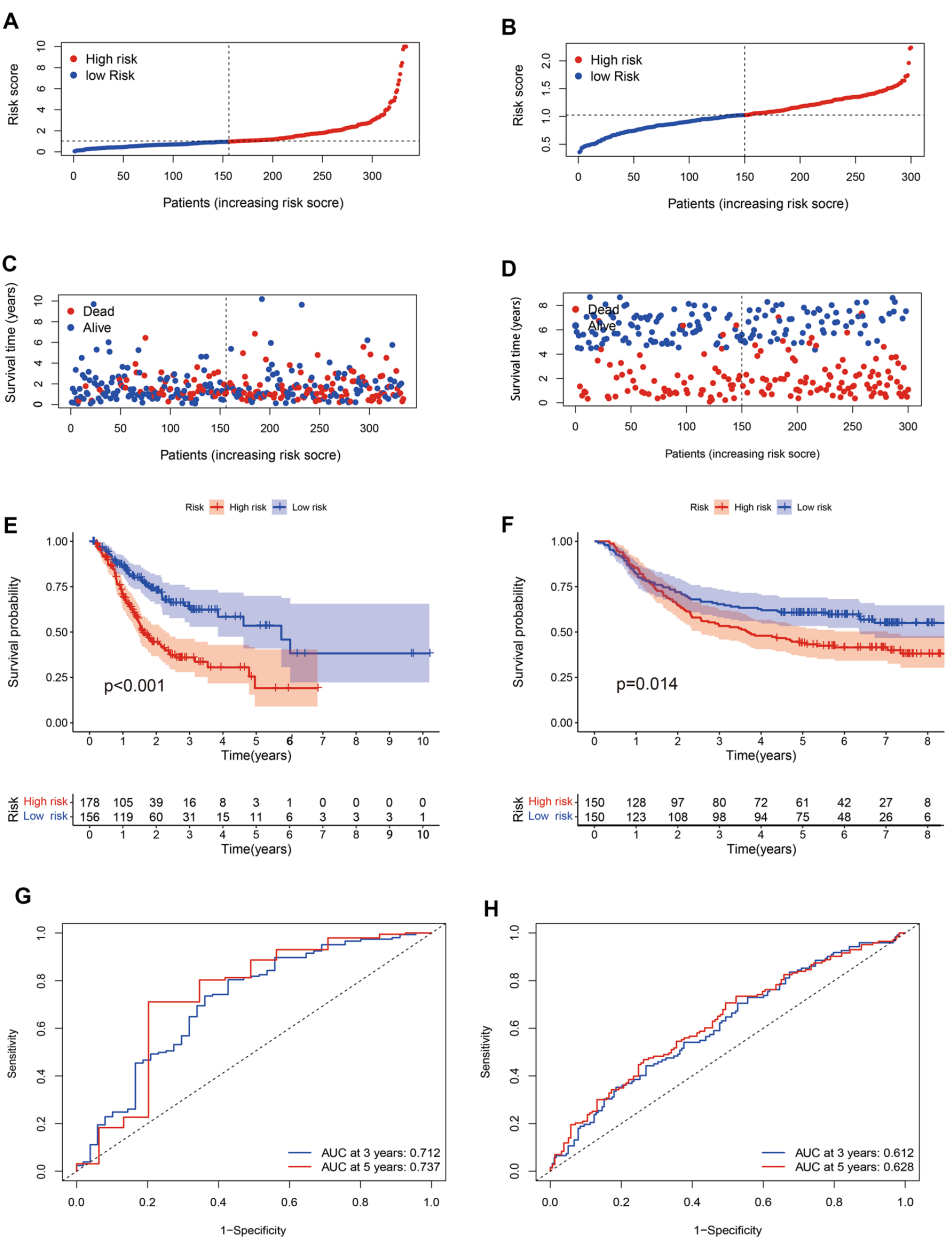
Construction and validation of an OSRLs signature. (**A,B**) The risk score curve in the training and validation cohort. The blue dot is patient with low-risk score, and the red dot is a patient with high-risk score. (**C,D**) The survival status curve in the training and validation cohort. The blue dots indicate survival, and the red dots indicate death. (**E,F**) The KM survival curve in the training and validation cohort. The blue is the survival curve of patients in the low-risk group, and the red is the survival curve of patients in the high-risk group. (**G,H**) The time-dependent receiver operating characteristic curve shows the 3-year and 5-year oxidative stress prediction accuracy of the oxidative stress-related lncRNAs in the training and validation cohort. Blue is the patient with 3-year ROC curve, and red is the patient with 5-year ROC curve.

We identified 102 oxidative stress-associated lncRNAs (Supplementary Table S6) by taking the intersection of 185 oxidative stress-associated DEGs and 13,978 lncRNAs from the UCSC dataset. The random survival forest algorithm showed that 33 lncRNAs interacted with oxidative stress (Supplementary Table S7). Multivariate analyses confirmed AC091057.1, TP53TG1, ARRDC1-AS1, SNHG5, DUXAP8, DIP2A-IT1, AL355001.1 and COLCA1 as OSRLs predictive signatures (Supplementary Table S8). The relationships between OSRLs and mRNA are shown in Supplementary Table S9. The risk score of each patient was calculated by the following formula: (− 0.375 $\times$ AC091057.1) + (0.439 $\times$ TP53TG1) + (− 0.335 $\times$ ARRDC1-AS1) + (0.020 $\times$ SNHG5) + (0.301 $\times$ DUXAP8) + (1.368 $\times$ DIP2A-IT1) + (− 1.387 $\times$ AL355001.1) + (− 0.209 $\times$ COLCA1). When the risk score was higher than the median value, the patient was classified as HRiG; otherwise, it was classified as LRiG. The survival status curve (Fig. 3A) and risk score curve (Fig. 3C) indicated clear differences in subgroup patients. The KM curve showed that HRiG patients had poorer overall survival than LRiG patients ﻿(*P*  < 0.01) (Fig. 3E). Moreover, the receiver operating characteristic (ROC) curve and area under the curve (AUC) value demonstrated that the risk model was sensitive and specific in GC prognosis prediction. The AUC values at 3 and 5 years reached 0.712 and 0.737 in the training cohort (Fig. 3G), respectively. In the validation set with similar results (Fig. 3B, D, F), the AUC values reached 0.612 and 0.628 at 3 years and 5 years, respectively (Fig. 3H).

### The prognostic value of the OSRLs signature

**Figure 4:**
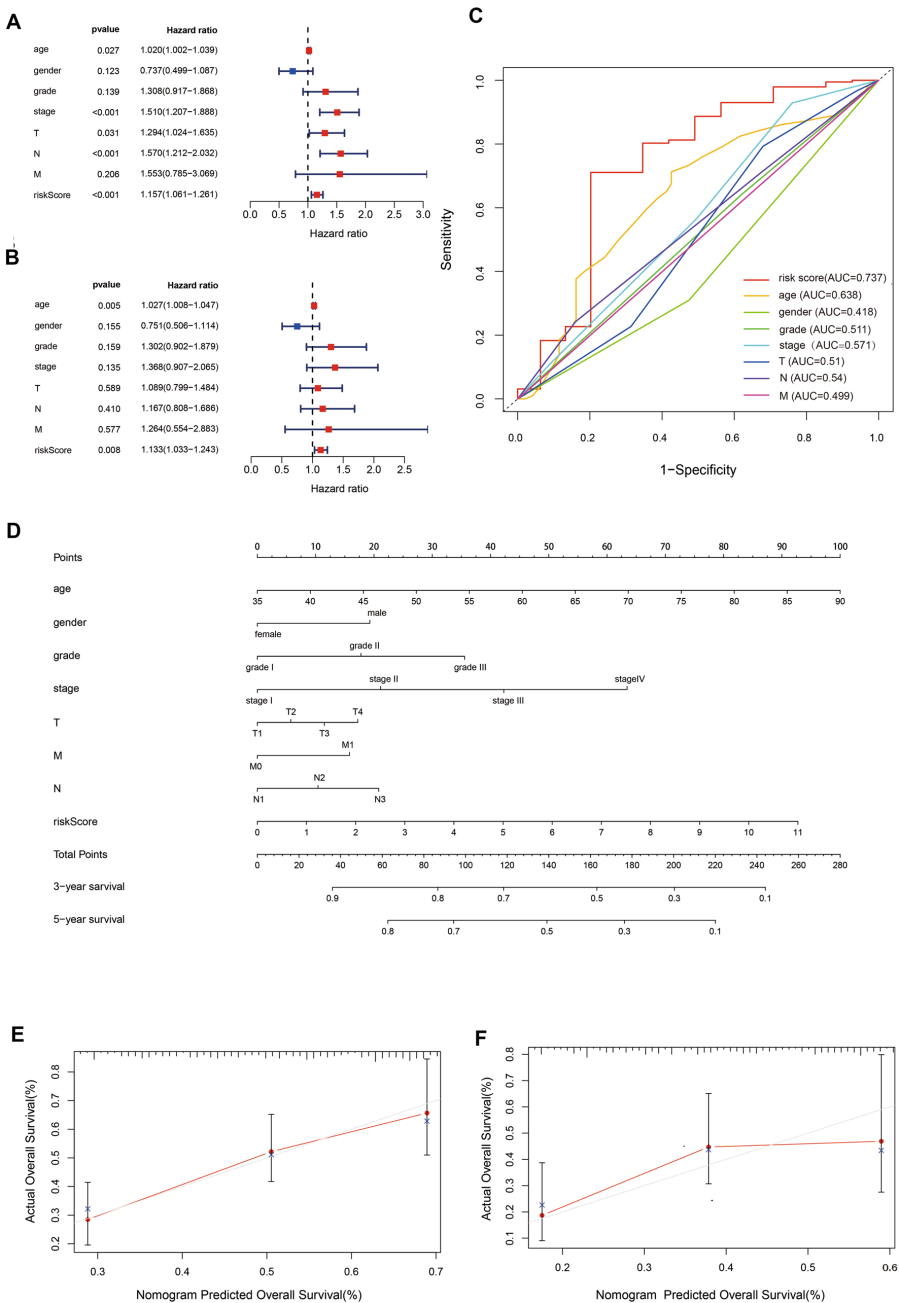
Prognostic value of the OSRLs signature. (**A,B**) Forest plots of OSRLs prognostic signature for univariate and multivariate Cox models. The red box represents significant prognostic features (*P* < 0.05). (**C**) Time-dependent receiver operating characteristic curves of the OSRLs signature and clinicopathological variables. The red line represents the time-dependent receiver operating characteristic curves of OSRLs features. (**D**) Construction of a nomogram containing 8 oxidative stress-related lncRNAs and clinicopathological features to predict the 3-year and 5-year overall survival of patients. (**E,F**) The calibration curve evaluates the accuracy of the nomogram in predicting 3-year and 5-year survival.

Notably, the risk model was confirmed as an independent prognostic characteristic in both univariate (HR = 1.156, 95% CI 1.061–1.261) and multivariate analyses (HR = 1.133, 95% CI 1.033–1.243) (Fig. 4A,B and Table 1), and the AUC value of the risk model reached 0.737 (Fig. 4C, Table 1). Later, we utilized the OSRLs signature to construct a prognostic nomogram to visualize the survival rate of each patient and evaluated the 3- and 5-year overall survival probabilities for GC patients (Fig. 4D). A calibration curve was utilized to evaluate the predictive performance of the nomogram. Figure 4E and F show that the predicted and true values of the calibration curve were linearly correlated. The above results indicated that the predictive signature and nomogram had good performance, accurate prediction and discriminative ability in GC prediction, which can play an important role in clinical management.

**Table 1:** Univariate and multivariate Cox analyses and AUC values of eight clinical indicators in GC patients.

Indicators	Univariate cox analysis		Multivariate cox analysis		AUC value
	HR (95% CI)	*P* value	HR (95% CI)	*P* value	
Age	1.020 (1.002, 1.039)	0.027	1.027 (1.008, 1.047)	0.005	0.638
Gender	0.737 (0.499, 1.087)	0.123	0.751 (0.506, 1.114)	0.155	0.418
Grade	1.308 (0.917, 1.868)	0.138	1.302 (0.902, 1.879)	0.159	0.511
Stage	1.510 (1.207, 1.888)	< 0.001	1.368 (0.907, 2.065)	0.135	0.571
T	1.294 (1.024, 1.635)	0.030	1.089 (0.799, 1.484)	0.589	0.510
N	1.569 (1.212, 2.032)	< 0.001	1.167 (0.808, 1.686)	0.410	0.543
M	1.552 (0.785, 3.069)	0.205	1.264 (0.554, 2.883)	0.577	0.499
Risk score	1.156 (1.061, 1.261)	< 0.001	1.133 (1.033, 1.243)	0.005	0.737

### Gene set enrichment analysis of the OSRLs signature

**Figure 5:**
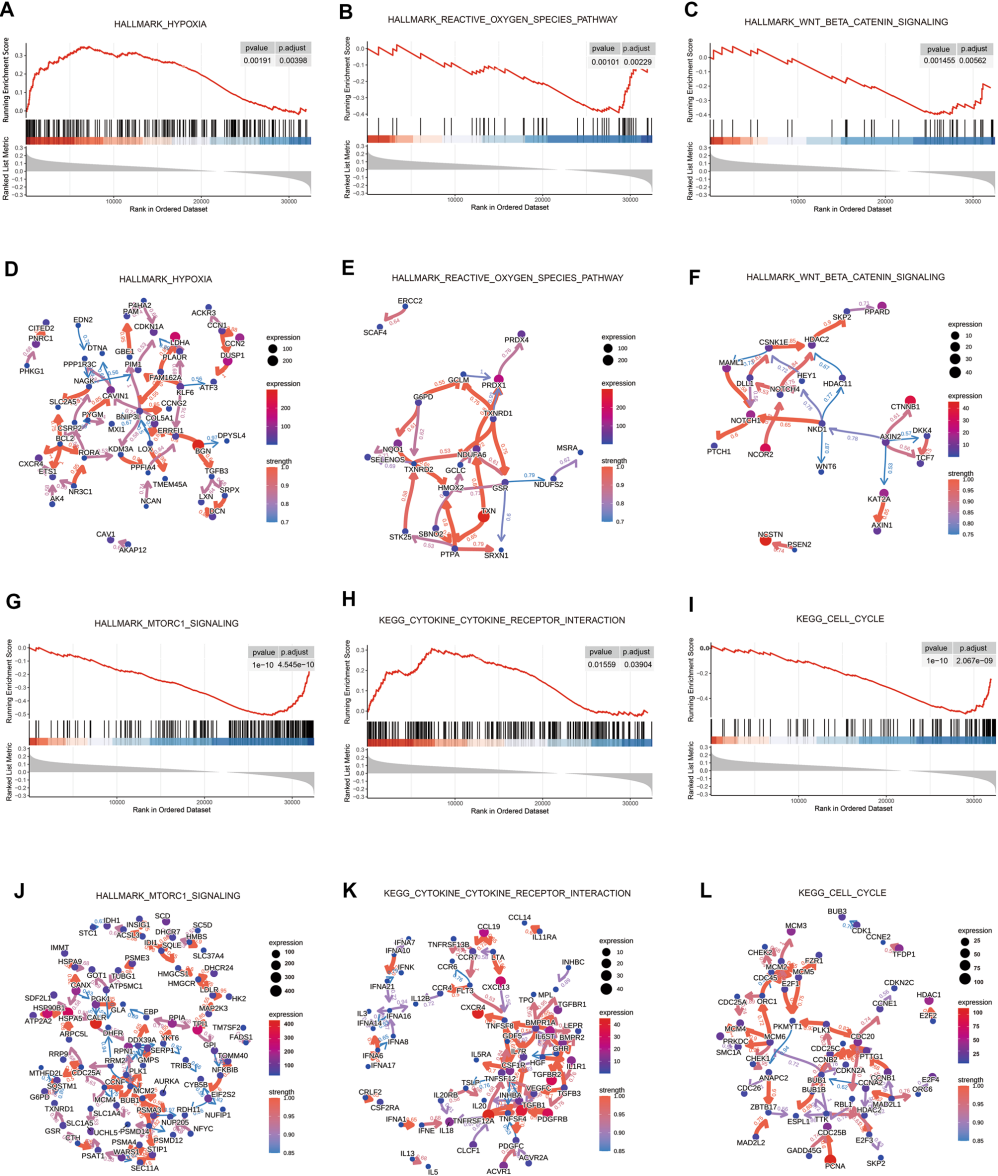
Pathway enrichment analysis of the OSRLs signature by GSEA. (**A,D**) Hallmark hypoxia and visualization of gene regulatory relationships in the pathway. (**B,E**) Hallmark reactive oxygen species pathway and visualization of gene regulatory relationships in pathway. (**C,F**) Hallmark Wnt β-catenin signaling and visualization of gene regulatory relationships in pathway. (**G,J**) Hallmark MTORC1 signaling and visualization of gene regulatory relationships in pathway. (**H,K**) KEGG cytokine‒cytokine receptor interaction and visualization of gene regulatory relationships in pathway. (**I,L**) KEGG cell cycle and visualization of gene regulatory relationships in pathway.

Gene enrichment analysis of predictive signature showed that the LRiG and HRiG patients were mainly related enriched in oxidative stress and immune pathways. For instance, the hallmark hypoxia (Fig. 5A,D) represented genes upregulated in response to low oxygen levels, and these genes were enriched in the HRiG. The hallmark reactive oxygen species pathway (Fig. 5B) was enriched in the LRiG; the related genes include the key gene thioredoxin (TXN) (Fig. 5E), which together with other enzymes constitutes scavenging enzyme systems involved in regulating mitochondrial ROS and protecting cells from oxidative stress^18^. As an upstream gene of ubiquinone oxidoreductase subunit A6 (NDUFA6), TXN may affect mitochondrial fitness by regulating the expression of NDUFA6^19^. The hallmark Wnt β-catenin signaling (Fig. 5C,F) and hallmark MTORC1 signaling (Fig. 5G,J) pathways were the significantly enriched immune-associated pathways in the LRiG. In addition, KEGG pathway analysis via GSEA showed enrichment in cytokine‒cytokine receptor interaction (Fig. 5H,K) and cell cycle (Fig. 5I,L) in HRiG and LRiG patients, respectively.

### Tumor immune microenvironment analysis

**Figure 6:**
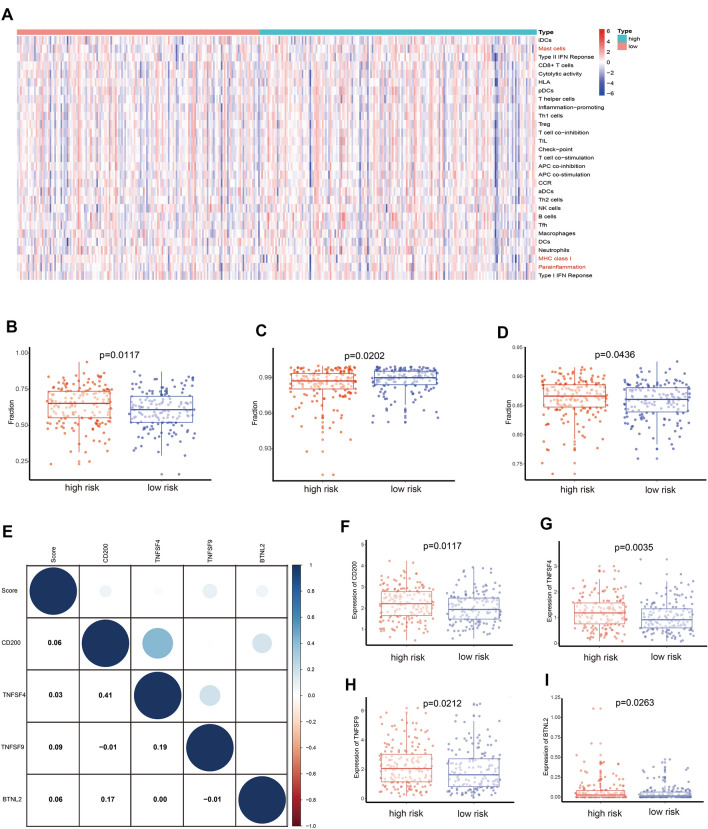
Prediction of immune cell infiltration based on the OSRLs signature. (**A**) A heatmap showing the association of immune cell infiltration levels with the OSRLs signature score between the low-risk groups and high-risk groups. (**B–D**) The level of immune cell infiltration significantly differences in low-risk group and high-risk group. The blue dots are low-risk group, and red dots are high-risk group. (**E**) The heatmap indicts the interaction between the risk score and immune checkpoints. (**F–I**) The expression levels of CD200, TNFSF4, TNFSF9, and BTNL2 were significant difference in the low-risk group and high-risk group. The blue dots are low-risk group, and the red dots are high-risk group.

**Figure 7:**
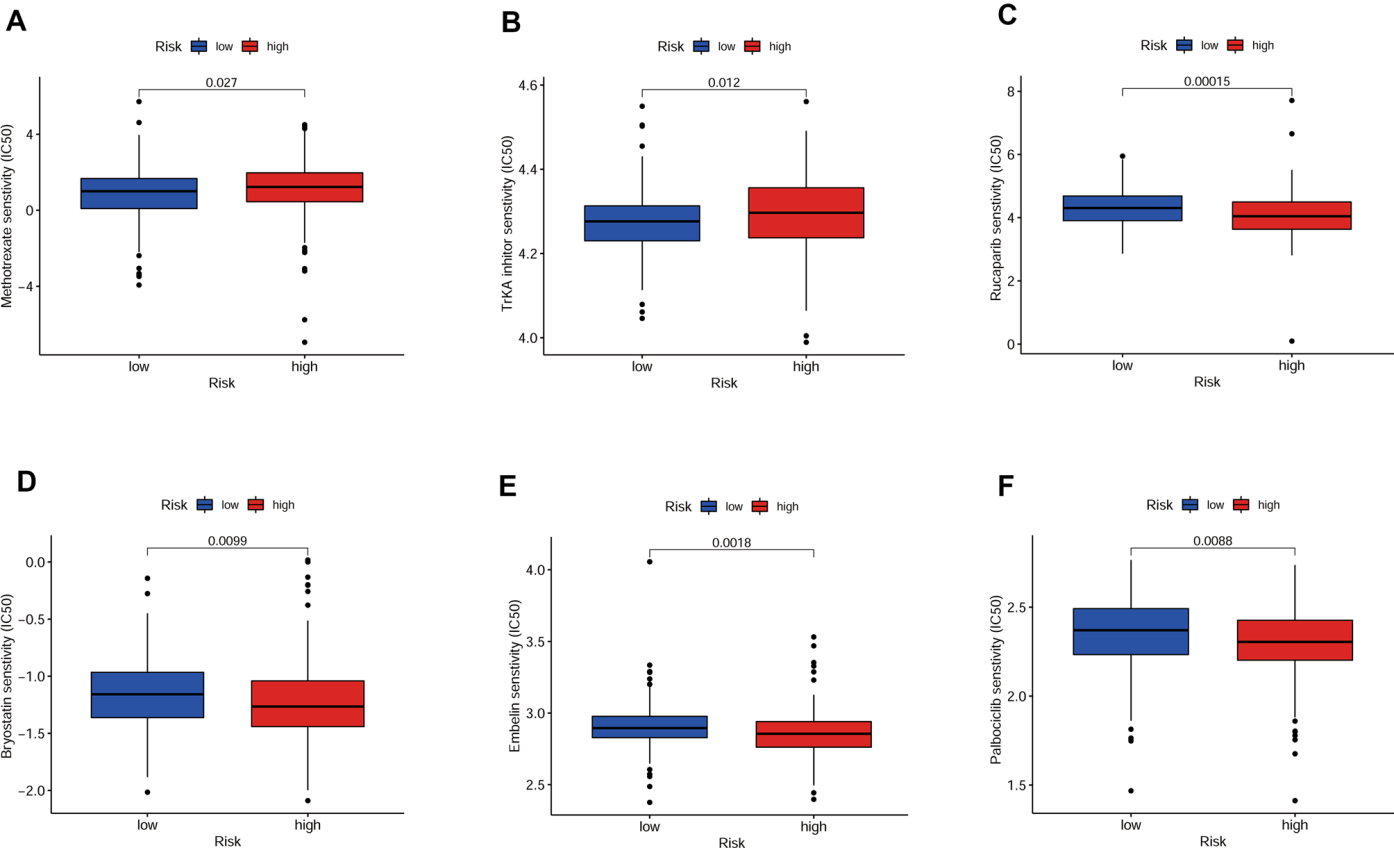
Prediction of chemotherapeutic drug sensitivity based on the OSRLs signature. (**A,B**) The boxplot showing the IC_50_ of the chemotherapy drugs methotrexate and TrKA inhibitors were more sensitive to high-risk group. (**C–F**) The chemotherapy drugs rucaparib, bryostatin, embelin, and palbociclib were more sensitive to low-risk group. The blue is low-risk group, and the red is high-risk group.

To confirm the interaction between OSRLs and the tumor immune microenvironment, we utilized the “ssGSEA” package to analyze differences in 16 immune-related cells and 13 immune-function scores in subgroups. As shown in Fig. 6A, mast cells, as cells involved in the immune response, were significantly differentially enriched between subgroups (*P* < 0.05) (Fig. 6B). Major histocompatibility complex class I (MHC class I) had higher expression levels in the LRiG than in the HRiG (*P* < 0.05) (Fig. 6C). This may be because MHC class I assist CD8 + T cells in eliminating malignant cells and providing long-term protective immunity. In contrast to the results for MHC class I, parainflammation, which is an inflammatory response by tissue cells under various stresses or abnormal functions, was more highly enriched in high-risk patients (*P* < 0.05) (Fig. 6D). Later, a heatmap was used to estimate the interaction between immune checkpoints and the risk model (Fig. 6E), and the expression of the CD200, TNFSF4, TNFSF9, and BTLN2 checkpoints was significantly increased in HRiG patients (Fig. 6F–I). Finally, the measurement standard for exploring the relationship between the OSRLs predictive signature and chemotherapy drugs (the half-maximal inhibitory concentration, IC_50_) was assessed. HRiG patients were more sensitive to methotrexate and TrKA inhibitors (Fig. 7A,B), while LRiG patients were more sensitive to rucaparib, bryostatin, embelin, and palbociclib (Fig. 7C–F).

### Experimental validation of oxidative stress-related lncRNAs in GC cell lines

**Figure 8:**
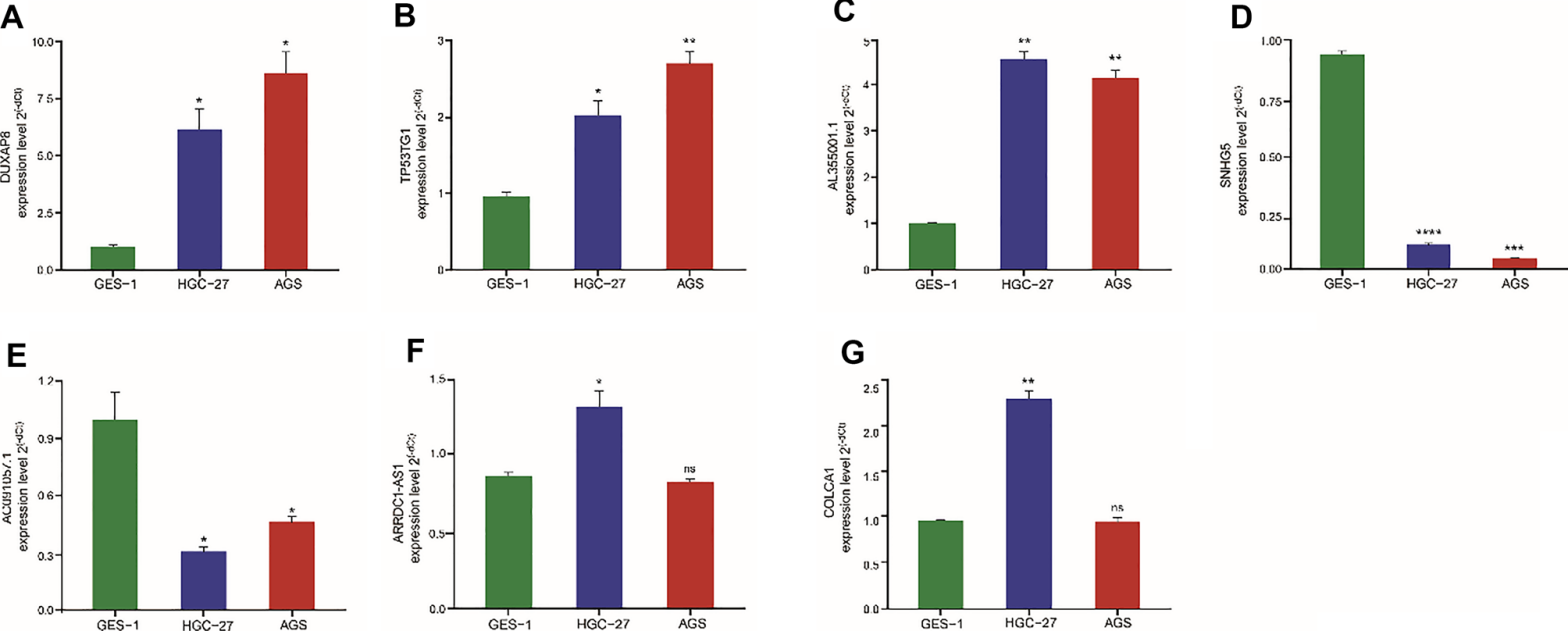
Comparison of the expression levels of 7 lncRNAs in GC cell lines. (**A–G**) The expression of 7 oxidative stress-related lncRNAs in normal (GES-1) and STDA cell lines. *P < 0.05, **P < 0.01, ns no significant. (**A**) DUXAP8, (**B**) TP53TG1, (**C**) AL355001.1, (**D**) SNHG5, (**E**) AC091057.1, (**F**) ARRDC1-AS1, (**G**) COLCA1.

The relative expression of 8 lncRNAs was verified by qRT‒PCR experiments among AGS, HGC-27, and GES-1 cells line. As shown in Fig. 8A–G, DUXAP8, TP53TG1, and AL355001.1 were significantly upregulated, while AC091057.1 and SNHG5 were significantly downregulated in AGS and HGC-27 cells compared with GES-1 cells. ARRDC1-AS1 and COLCA1 were upregulated in HGC-27 cells, and there was no significant difference in AGS cells compared with GES-1 cells. DIP2A-IT1 expression was not significantly different among the GES-1, AGS, and HGC-27 cell lines.

## Discussion

GC is the third most common cause of cancer-related death in the world. Its 5-year survival rate is only 20% to 30%, indicating that it poses serious harm to physical health. Previous studies have shown that tumor cells produce more ROS than normal cells due to mitochondrial and metabolic disorders. To prevent the occurrence and metastasis of cancer, antioxidant therapy targeting GC may be a new approach to the treatment of GC^20^. However, only a few studies have focused on the prognostic characteristics of oxidative stress. In contrast, more studies have focused on the mechanism and treatment of oxidative stress in GC. Some therapeutic approaches mostly target only tumor cells with little attention to the tumor microenvironment (TME). With the rapid development of bioinformatics technology, there is increasing evidence that lncRNAs contribute to carcinogenesis and tumor development and can be used as a predictive feature associated with oxidative stress^21^. However, no report has been published about the prognostic significance of OSRLs in GC. Therefore, it is imperative to select oxidative stress-related biomarkers involved in GC development, construct a more efficient risk prediction model for GC, improve the prognosis of GC and seek new targets for microenvironment-targeted therapy.

In this study, we identified 185 oxidative stress-related DEGs between the TCGA-STAD dataset and oxidative stress-related gene set by bioinformatics methods. Next, to identify enriched pathways of oxidative stress-related DEGs, we used GO and KEGG pathway analysis and integrated these results, yielding two gene sets. The GO set of DEGs was significantly enriched in “response to oxidative stress” (*P* = 8.82 $\times\, {10}^{-30}$) in the BP category, which is consistent with the research direction in this study. The top three terms of the remaining set were enriched in “lipid and atherosclerosis” (*P* = 1.50 $\times\, {10}^{-9}$), “drug metabolism-cytochrome P450” (*P* = 4.79 $\times\, {10}^{-9}$), and “IL-17 signaling pathway” (*P* = 2.89 $\times\, {10}^{-10}$). In the KEGG analysis, atherosclerosis, as a chronic inflammatory disease, was found to be affected by the state of oxidative stress, and ROS was found to play a crucial role in inflammatory responses, cell growth, apoptosis, and vascular homeostasis, all of which may be relevant to cancer^22^. Cytochrome P450 (CYP450) is a hemoglobin superfamily that plays a significant role in drug detoxification, cellular metabolism, and homeostasis^23^. In this family, CPY2E1 was found to be involved in the occurrence and development of liver cancer because it can generate high levels of ROS^24^. The final term “IL-17 signaling pathway” has been verified to promote oxidative stress-induced hepatocyte apoptosis through the Nrf2/keap1 signaling pathway^25^. This indicated that oxidative stress-related genes were activated by the IL-7 signaling pathway, which influenced GC progression^26^. The mechanism of oxidative stress-related genes in GC still needs to be further explored.

A previous study indicated that lncRNAs play a significant role in the oxidative stress of cancer patients. Wang et al. indicated that lncRNA H19 and HULC activated oxidative stress by H_2_O_2_ and glucose oxidase to regulate CCA cell migration and invasion^27^. Similarly, researchers have indicated that lncRNA NEAT1 is upregulated by (-)-epigallocatechin-3-gallate (EGCG)-induced oxidative stress, increasing cisplatin intake in lung cancer treatment^28^. However, the explanation of lncRNAs interaction with oxidative stress in cancer, especially in GC was awfully insufficient are unclear. In this regard, considering the abundance of oxidative stress-related genes, Pearson correlation analysis was used to explore OSRLs. Then, the RSF algorithm was exploited to discriminate OSRLs and obtained a total of 34 lncRNAs related to prognosis in GC. Finally, Cox regression was used to identify DUXAP8, TP53TG1, SNHG5, AC091057.1, AL355001.1, ARRDC1-AS1, DIP2A-IT1, and COLCA1 to construct the prognostic signature of OSRLs. Among them, DUXAP8 can promote GC development by epigenetically repressing PLEKHO1 expression by binding EZH2 and SUZ12^29^. Similarly, lncRNA TP53TG1 inhibits the activation of the PI3K/Akt signaling pathway by binding to the human tumor protein CIP2A, which leads to the inhibition of GC cell proliferation and survival^30^. Unlike the first two lncRNAs, the role of SNHG5 in tumors varies depending on gene copy variation (deletion or amplification), transcription factors, histone modification, or DNA methylation differences in gastric patients, it can either promote or suppress tumor growth^31^. In the study of glioma, SNHG5 promotes tumor growth by targeting E2F3, and E2F3 and E2F1 are both transcription factors of the E2Fs encoding gene family and are associated with poor prognosis of gastric cancer. Current study shows that E2F1 interacts with ARRDC1-AS1, so we infer that ARRDC1-AS1 has a similar function to SNHG5, and promotes the growth of gastric cancer cells by targeting E2F1. The remaining 4 lncRNAs (COLCA1, AC091057.1, AL355001.1, and DIP2A-IT1) have no relevant research in cancer. Therefore, it is necessary to verify the function of 8 lncRNAs through experiments, to determine the potential of OSRLs as prognostic biomarkers of GC that understand the mechanism of GC to develop therapeutic drugs for GC.

DUXAP8 is a biomarker and therapeutic target of various cancers that is upregulated in GC, promotes cell proliferation and migration, and then accelerates the development of GC^32^, and this study verified the upregulation of DUXAP8 in GC. Previous studies have shown that lncRNA TP53TG1 can not only inhibit the development of GC by regulating the stability of CIP2A34^30^, but also play a promoting role in cancer development. For example, TP53TG1 increased the sensitivity of non-small cell lung cancer cells to DNA damaging agents by regulating the miR-18a/PTEN axis^33^. Interestingly, the qRT‒PCR results of this study also showed that lncRNA TP53TG1 was highly expressed in GC and could be a potential risk factor rather than a protective factor. Similarly, lncRNA SNHG5 has been found to promote tumorigenesis and metastasis in a variety of cancers, while other studies have indicated that it suppresses tumorigenesis^34^. In the present study, SNHG5 was upregulated in GC cells, indicating its role as a risk factor. However, some studies have also shown that the expression of lncRNA SNHG5 was lower in GC cells than in healthy cells and benign gastric disease cells^35^. DIP2A-IT1 was upregulated in the analysis of DEGs in osteosarcoma tissue transcripts. But, in the present study, the expression of lncRNA DIP2A-IT1 was not significantly different between the GC group and normal gastric cells. Then, we found 4 novel lncRNA biomarkers in GC and verified them experimentally. One of them was the prognostic protective factor lncRNA AC091057.1, which was associated with 68.87% of mRNAs in our study. The expression of AC091057.1 was experimentally verified and was consistent with the results of a pancreatic cancer study indicating it as a protective factor in cancer^36^. In a diffuse large B cell lymphoma (DLBCL) study, the knockdown of ARRDC1-AS1 exacerbated proliferation, inhibited apoptosis, and promoted invasion and migration^37^. Therefore, we presumed that the high expression of ARRDC1-AS1 in GC indicates its role as a tumor suppressor. In another study of coronary artery endothelial cells, the high expression of COLCA1 was stimulated by oxidized low-density lipoprotein, which regulates the level of oxidative stress in cells, thus leading to a sustained inflammatory response in cells^38^. This was consistent with the high expression of COLCA1 in our experiment. The remaining lncRNA, AL355001.1, was detected in recent years, and no relevant experiment has proven its expression in cancer cells. Our study was the first to experimentally verify the expression of AL355001.1, which was higher in GC cell lines than in normal cell lines. Overall, the results indicate that these 8 lncRNAs can be used as prognostic factors for GC patients, consistent with our bioinformatics analysis results.

Increasing evidence has shown that oxidative stress plays an important role in the tumor immune microenvironment. As highlighted by Wu et al. ROS restricts the cytosolic translocation of SUMO-specific protease 7 and affected the metabolism and functional activity of CD8 + T cells, which weakens antitumor activity in vivo^39^. To confirm the enriched pathways, we used GSEA of genes enriched in the HRiG and LRiG and integrated those results, yielding two aspects. One side indicated that tumor hallmarks were associated with ROS in both the HRiG and the LRiG. For example, the Hallmark hypoxia and Hallmark reactive oxygen species pathways were closely associated with the oxidative stress-related signature in this study. On the other hand, GSEA showed that cytokine‒cytokine receptor interaction, cell cycle, Wnt/-$\beta$ catenin signaling, and mTORC1 signaling were strongly connected to antitumor immunity and decreased oxidative stress in GC. Based on this, we speculated that antitumor immunity and oxidative stress were closely related in GC. Previous studies have shown that cytokines enhance the expansion and persistence of CAR-T cells and enhance their function in the immunosuppressive TME^40^. In addition, the Wnt pathway is an upstream pathway that participates in the regulation of the cell cycle, tumor, and other pathways^41^. The canonical Wnt signaling pathway is activated by the mitotic CDK14/cyclin Y complex via phosphorylation of the LRP6 coreceptor, which leads to anti-inflammatory signaling that inhibits tumor growth^42^. Moreover, mTOR is located at the core of tumor-related signaling pathways^43^ and plays a key regulatory role in the cell cycle^44^. The importance of mTORC1 in regulating innate and adaptive immunity has been widely recognized; for example, it regulates immune tolerance related to regulatory T cells^45^. Overall, we found a strong association between the predictive signature and antitumor immunity, providing new biomarkers for cancer immunotherapy.

Immunomodulation has been indicated to play an important role in cancer treatment. Immune cell infiltration can seriously influence cancer progression and the response to immunotherapy and is related to the prognosis of cancer patients. Patients with obvious tumor clinical manifestations are usually in the tumor escape phase; that is, tumor cells with reduced immunogenicity grow to a certain extent and surpass the ability of the body's immune response to avoid an antitumor immune response^46^. This may affect the levels of immunosuppressive and immune response cells. Therefore, we predicted a significant difference in immunotherapy in HRiG/LRiG patients. The outcomes showed that mast cells were prominent in the HRiG, so the mast cells might directly suppress immunity by promoting angiogenesis and the infiltration of mast cell subsets to facilitate different degrees of tumor development^47^. Immune checkpoint blockade, as one of the methods of immunotherapy, has been used to improve the prognosis of patients with malignant tumors^48^. The expression levels of CD200, TNFSF4, TNFST9, and BTNL2 were higher in HRiG patients, and these patients may benefit from immune checkpoint blockade to enhance the immune response or inhibit oxidative stress, which could improve prognosis in the HRiG. In recent years, some studies have reported that TPE-DPA-TCyp^49^, CPT^50^, MitoCAT-g^51^, TSEOP^52^ are useful as polymer prodrugs or nano-delivery systems of antitumor immune drugs to modulate tumor oxidative stress. These findings regarding oxidative stress provide a new idea for tumor immunotherapy and a safe and economical method for the treatment of tumor patients.

In general, OSRLs, which were validated by qRT‒PCR experiments, have the potential to be biomarkers for predicting the overall survival rate of STAD patients. Notably, the signature might regulate immune infiltration levels as well as immune function. Therefore, the mechanisms and relationships among oxidative stress, lncRNAs, immunity, and GC deserve further exploration and validation. We believe that the 8 OSRLs signature can guide research on the biological behavior of GC and its clinical prognosis. However, there are some limitations to this study: (1) The study used one external validation set, and more external validation sets are required to ensure the validity of the model. (2) It was necessary to further verify the mechanism of OSRLs in GC by performing experiments.

## Supplementary Information

Supplementary Tables.

## Data Availability

Publicly available datasets were analyzed in this study. This data can be found here: TCGA database (http://www.cancer.gov/tcga), GEO database (https://www.ncbi.nlm.nih.gov/geo/), GeneCards (https://www.genecards.org/), and UCSC Xena database (https://xenabrowser.net/).

## Abbreviations

GC: Gastric cancer
LncRNAs: Long non-coding RNAs
TCGA: The cancer genome atlas
GEO: Gene expression omnibus
OSRLs: Oxidative stress related lncRNAs
ROC: Receiver operating characteristic
ROS: Reactive oxygen species
DEGs: Differentially expressed genes
GO: Gene ontology
KEGG: Kyoto encyclopedia of genes and genomes
RSF: Random survival forest
HRiG: High-risk group
LRiG: Low-risk group
TNM: Tumor node metastasis
IC_50_: The half-maximal inhibitory concentration
GES-1: Gastric epithelial cell line
GSEA: Gene set enrichment analysis

## References

[1] Smyth EC, Nilsson M, Grabsch HI (2020). DUXAP8, a pseudogene derived lncRNA, promotes growth of pancreatic carcinoma cells by epigenetically silencing CDKN1A and KLF21. Gastric Cancert.

[2] Sexton RE, Al Hallak MN, Diab M, Azmi AS (2020). Gastric cancer: A comprehensive review of current and future treatment strategies. Cancer Metastasis Rev..

[3] Galluzzi L, Humeau J, Buqué A (2020). Immunostimulation with chemotherapy in the era of immune checkpoint inhibitors. Nat. Rev. Clin. Oncol..

[4] Xu Y, Zhang P, Zhang K, Huang C (2021). The application of CA72–4 in the diagnosis, prognosis, and treatment of gastric cancer. Biochem. Biophys. Acta..

[5] Thrift AP, El-Serag HB (2020). Burden of gastric cancer. Clin. Gastroenterol. Hepatol..

[6] Renaudin X (2021). Reactive oxygen species and DNA damage response in cancer. Int. Rev. Cell Mol. Biol..

[7] Sies H, Jones DP (2020). Reactive oxygen species (ROS) as pleiotropic physiological signalling agents. Nat. Rev. Mol. Cell Biol..

[8] Kim J, Cho SY, Cho D (2017). Oxidative stress and FoxO transcription factors in cardiovascular aging. Curr. Med. Chem..

[9] Terado T, Kim CJ, Ushio A (2022). Cryptotanshinone suppresses tumorigenesis by inhibiting lipogenesis and promoting reactive oxygen species production in KRAS-activated pancreatic cancer cells. Int. J. Oncol..

[10] Huang H, Chen Y, Yin N (2023). Unsaturated fatty acid liposomes selectively regulate glutathione peroxidase 4 to exacerbate lipid peroxidation as an adaptable liposome platform for anti-tumor therapy. Mol. Pharm..

[11] Xing C, Sun SG, Yue ZQ, Bai F (2021). Role of lncRNA LUCAT1 in cancer. Biomed. Pharmacotherap..

[12] Qi W, Li Z, Xia L (2019). LncRNA GABPB1-AS1 and GABPB1 regulate oxidative stress during erastin-induced ferroptosis in HepG2 hepatocellular carcinoma cells. Sci. Rep..

[13] Pu Y, Tan Y, Zang C (2021). LAMTOR5-AS1 regulates chemotherapy-induced oxidative stress by controlling the expression level and transcriptional activity of NRF2 in osteosarcoma cells. Cell Death Dis..

[14] Zhang L, Kang W, Lu X (2018). LncRNA CASC11 promoted gastric cancer cell proliferation, migration and invasion in vitro by regulating cell cycle pathway. Cell Cycle.

[15] Lv Y, Wang Y, Song Y (2021). LncRNA PINK1-AS promotes Gαi1-driven gastric cancer tumorigenesis by sponging microRNA-200a. Oncogene.

[16] Zhao Y, Liu Y, Lin L (2018). The lncRNA MACC1-AS1 promotes gastric cancer cell metabolic plasticity via AMPK/Lin28 mediated mRNA stability of MACC1. Mol. Cancer.

[17] Sato N, Tamada Y, Yu G, Okuno Y (2022). CBNplot: Bayesian network plots for enrichment analysis. Bioinformatics (Oxford, England).

[18] Su L, Zhang J, Gomez H (2022). Mitochondria ROS and mitophagy in acute kidney injury. Autophagy.

[19] Castracani CC, Longhitano L, Distefano A (2020). Role of 17β-estradiol on cell proliferation and mitochondrial fitness in glioblastoma cells. J. Oncol..

[20] Lv J, Xie M, Zhao S (2021). Nestin is essential for cellular redox homeostasis and gastric cancer metastasis through the mediation of the Keap1-Nrf2 axis. Cancer Cell Int..

[21] Zhang M, Du G, Li Z (2022). An oxidative stress-related genes signature for predicting survival in bladder cancer: Based on TCGA database and bioinformatics. Int. J. Gen. Med..

[22] Kattoor AJ, Pothineni NVK, PalagiriMehta DJL (2017). Oxidative stress in atherosclerosis. Curr. Atheroscler. Rep..

[23] Song YS, Annalora AJ, Marcus CB (2022). Cytochrome P450 1B1: A key regulator of ocular iron homeostasis and oxidative stress. Cells.

[24] Massart J, Begriche K, Hartman JH, Fromenty B (2022). Role of mitochondrial cytochrome P450 2E1 in healthy and diseased liver. Cells.

[25] Xu X, Zhang S, Song X (2018). IL-17 enhances oxidative stress in hepatocytes through Nrf2/keap1 signal pathway activation. Int. J. Clin. Exp. Pathol..

[26] Marković I, Savvides SN (2020). Modulation of signaling mediated by TSLP and IL-7 in inflammation, autoimmune diseases, and cancer. Front Immunol..

[27] Wang WT, Ye H, Wei PP (2016). LncRNAs H19 and HULC, activated by oxidative stress, promote cell migration and invasion in cholangiocarcinoma through a ceRNA manner. J. Hematol. Oncol..

[28] Chen A, Jiang P, Zeb F (2020). EGCG regulates CTR1 expression through its pro-oxidative property in non-small-cell lung cancer cells. J. Cell Physiol..

[29] Ma HW, Xie M, Sun M (2017). The pseudogene derived long noncoding RNA DUXAP8 promotes gastric cancer cell proliferation and migration via epigenetically silencing PLEKHO1 expression. Oncotarget.

[30] Fang D, Ou X, Sun K (2022). m6A modification-mediated lncRNA TP53TG1 inhibits gastric cancer progression by regulating CIP2A stability. Cancer Sci..

[31] Han W, Shi J, Cao J (2020). Latest advances of long non-coding RNA SNHG5 in human cancers. Onco. Targets Ther..

[32] Xue C, Cai X, Jia J (2021). Long non-coding RNA double homeobox a pseudogene 8: A novel oncogenic propellant in human cancer. Front. Cell Dev. Biol..

[33] Xiao H, Liu Y, Liang P (2018). TP53TG1 enhances cisplatin sensitivity of non-small cell lung cancer cells through regulating miR-18a/PTEN axis. Cell Biosci..

[34] Li YH, Hu YQ, Wang SC (2020). LncRNA SNHG5: A new budding star in human cancers. Gene.

[35] Li X, Du Y, Wang Y (2021). The value of LncRNA SNHG5 as a marker for the diagnosis and prognosis of gastric cancer. Am. J. Transl. Res..

[36] Jiang W, Du Y, Zhang W, Zhou W (2022). Construction of a prognostic model based on cuproptosis-related lncRNA signatures in pancreatic cancer. Can. J. Gastroenterol. Hepatol..

[37] Xu H, Yu X, Yang Z (2021). PAX5-activated lncRNA ARRDC1-AS1 accelerates the autophagy and progression of DLBCL through sponging miR-2355–5p to regulate ATG5. Life Sci..

[38] Li MP, Hao ZC, Yan MQ (2022). Possible causes of atherosclerosis: lncRNA COLCA1 induces oxidative stress in human coronary artery endothelial cells and impairs wound healing. Ann. Transl. Med..

[39] Wu Z, Huang H, Han Q (2022). SENP7 senses oxidative stress to sustain metabolic fitness and antitumor functions of CD8+ T cells. J. Clin. Invest..

[40] Bell M, Gottschalk S (2021). Engineered cytokine signaling to improve CAR T cell effector function. Front. Immunol..

[41] Bugter JM, Fenderico N, Maurice MM (2021). Mutations and mechanisms of WNT pathway tumour suppressors in cancer. Nat. Rev. Cancer.

[42] Vallée A, Lecarpentier Y, Vallée JN (2019). Targeting the canonical WNT/β-catenin pathway in cancer treatment using non-steroidal anti-inflammatory drugs. Cells.

[43] Gargalionis AN, Papavassiliou KA, Basdra EK, Papavassiliou AG (2022). mTOR signaling components in tumor mechanobiology. Int. J. Mol. Sci..

[44] Xie Z, Li W, Ai J (2022). C2orf40 inhibits metastasis and regulates chemo-resistance and radio-resistance of nasopharyngeal carcinoma cells by influencing cell cycle and activating the PI3K/AKT/mTOR signaling pathway. J. Transl. Med..

[45] Do MH, Wang X, Zhang X (2020). Nutrient mTORC1 signaling underpins regulatory T cell control of immune tolerance. J. Exp. Med..

[46] Lin C, He H, Liu H (2019). Tumour-associated macrophages-derived CXCL8 determines immune evasion through autonomous PD-L1 expression in gastric cancer. Gut.

[47] Sammarco G, Varricchi G, Ferraro V (2019). Mast cells, angiogenesis and lymphangiogenesis in human gastric cancer. Int. J. Mol. Sci..

[48] Kwon M, An M, Klempner SJ (2021). Determinants of response and intrinsic resistance to PD-1 blockade in microsatellite instability-high gastric cancer. Cancer Discov..

[49] Chen C, Ni X, Jia S (2019). Massively evoking immunogenic cell death by focused mitochondrial oxidative stress using an AIE luminogen with a twisted molecular structure. Adv. Mater..

[50] Yin W, Ke W, Chen W (2019). Integrated block copolymer prodrug nanoparticles for combination of tumor oxidative stress amplification and ROS-responsive drug release. Biomaterials.

[51] Gong N, Ma X, Ye X (2019). Carbon-dot-supported atomically dispersed gold as a mitochondrial oxidative stress amplifier for cancer treatment. Nat. Nanotechnol..

[52] Ma S, Song W, Xu Y (2020). Rationally designed polymer conjugate for tumor-specific amplification of oxidative stress and boosting antitumor immunity. Nano Lett..

